# In vitro assembly of xenotropic murine leukemia virus-related virus CA-NC protein

**DOI:** 10.1186/1742-4690-8-S1-A236

**Published:** 2011-06-06

**Authors:** Romana Hadravová, Jitka Štokrová, Michal Doležal, Iva Pichová, Tomáš Ruml, Michaela Rumlová

**Affiliations:** 1 Department of Biochemistry, Institute of Organic Chemistry and Biochemistry Czech Academy of Sciences, Prague, Czech Republic; 2 Department of Biochemistry and Microbiology, Institute of Chemical Technology, Prague, Czech Republic

## 

Using in vitro expression/assembly system we studied the formation of virus-like particles Xenotropic Murine Leukemia Virus-related virus (XMRV). XMRV is novel human gammaretrovirus discovered in association with human prostate tumors. The genome organization is typical for gammaretroviruses consisting of two overlapping ORFs coding for Gag-Pro-Pol and Env polyproteins. The predicted Gag polyprotein consists of 536 amino acids and is separated from the Pro-Pol sequence by UAG stop codon.

Based on the amino acids similarities between MLV and XMRV Gag polyproteins, we designed primers bordering CA-NC region of Gag. Resulting PCR fragment was cloned into pET22b vector for expression of CA-NC in E. coli. We found that purified XMRV full-length CANC, starting with the conserved proline residue at the N-terminus of CA, was not able to assemble into particles. However, a modification of the N-terminus of CANC (modCANC) enabled formation of spherical particles. Moreover, the negative staining of the in vitro assembled particles of XMRV modCANC revealed different organization of protein layers in comparison to CA-NC of M-PMV.

